# Resonance phenomena controlled by external feedback signals and additive noise in neural systems

**DOI:** 10.1038/s41598-019-48950-3

**Published:** 2019-09-02

**Authors:** Sou Nobukawa, Natsusaku Shibata, Haruhiko Nishimura, Hirotaka Doho, Nobuhiko Wagatsuma, Teruya Yamanishi

**Affiliations:** 1 Department of Computer Science, Chiba Institute of Technology, 2-17-1 Tsudanuma, Narashino, Chiba, 275-0016 Japan; 2 Graduate School of Applied Informatics, University of Hyogo, 7-1-28 Chuo-ku, Kobe, Hyogo, 650-8588 Japan; 3 Faculty of Education, Teacher Training Division, Kochi University, 2-5-1 Akebono-cho, Kochi, 780-8520 Japan; 4 Faculty of Science, Department of Information Science, Toho University, 2-2-1 Miyama, Funabashi, Chiba, 274-8510 Japan; 5 AI & IoT Center, Department of Management and Information Sciences, Fukui University of Technology, 3-6-1 Gakuen, Fukui, Fukui, 910-8505 Japan

**Keywords:** Dynamical systems, Network models, Biological physics

## Abstract

Chaotic resonance is a phenomenon that can replace the fluctuation source in stochastic resonance from additive noise to chaos. We previously developed a method to control the chaotic state for suitably generating chaotic resonance by external feedback even when the external adjustment of chaos is difficult, establishing a method named reduced region of orbit (RRO) feedback. However, a feedback signal was utilized only for dividing the merged attractor. In addition, the signal sensitivity in chaotic resonance induced by feedback signals and that of stochastic resonance by additive noise have not been compared. To merge the separated attractor, we propose a negative strength of the RRO feedback signal in a discrete neural system which is composed of excitatory and inhibitory neurons. We evaluate the features of chaotic resonance and compare it to stochastic resonance. The RRO feedback signal with negative strength can merge the separated attractor and induce chaotic resonance. We also confirm that additive noise induces stochastic resonance through attractor merging. The comparison of these resonance modalities verifies that chaotic resonance provides more applicability than stochastic resonance given its capability to handle attractor separation and merging.

## Introduction

Over decades, many types of synchronization phenomena in nonlinear systems have been explored (reviewed in^1–3^). Among them, stochastic resonance, in which additive noise enhances the response to weak input signals, has been widely observed in nonlinear systems, such as global climate^4^, economic^5^, electric^6^, and biological^7–10^ systems. In particular, regarding recent studies about stochastic resonance in neural systems, we reported that spike-timing-dependent plasticity might be enhanced by stochastic resonance, and the enhancement depends on neural spiking patterns^11^. Likewise, Teramae *et al*.^12^ showed that spontaneous activity observed in cortical neural networks might be produced by the effect of stochastic resonance through a lognormal distribution of synaptic weights. Based on this study, we found that the temporal complexity of spontaneous activity produced by stochastic resonance depends on small-world networks^13^. Furthermore, stochastic resonance is influenced by the neural network structure^14–16^ (reviewed in^10^). For instance, Wang *et al*.^14^, Yilmaz *et al*.^15^, and Yu *et al*.^16^ demonstrated that in the stochastic resonance of neural systems, the presence of electrical synapses, synaptic delay, and scale freeness may promote signal transmission. Regarding the input signals in stochastic resonance, not only a periodic weak input signal but also more complex input signals have been used^17–19^ (reviewed in^10^). Gao *et al*. demonstrated that stochastic resonance can arise under envelope-modulated signals that are widely observed in regional neural activity, such as phase-amplitude coupling signals, and its presence is maximized by the optimal balance between excitatory and inhibitory neural populations^20^. Besides spike transmission, the mechanism of stochastic resonance might be applicable to higher brain functional levels (reviewed in^21,22^). For example, Garrett *et al*.^23,24^ and Mcintosh *et al*.^25^ demonstrated that the degree of fluctuations in neural activity observed from neuroimaging can reflect age, cognitive function, and recognition accuracy^23–25^.

Given the potential enhancement of signal sensitivity, applications of stochastic resonance have gained attention in biomedical engineering^26–30^. For instance, Kurita *et al*.^26,29^ proposed a wearable device utilizing the effect of stochastic resonance to implement surgical grasping forceps, enhancing human tactile sensory performance through vibration. Enders *et al*.^27^ and Seo *et al*.^28^ proposed a method to improve touch sensation in paralyzed patients and stroke survivors. Moreover, at cognitive levels of brain function, Van der Groen *et al*.^30^ developed a method to enhance perceptual decision-making by exploiting stochastic resonance. Specifically, the optimal amount of noise applied by transcranial random noise stimulation to the visual cortex conforms a non-invasive brain stimulation technique that enhances the accuracy of perceptual decisions.

A chaotic system exhibits various kinds of dynamical characteristics, such as intermittency chaos, hyperchaos, and bubbling transition^31,32^ (reviewed in^33,34^). Among them, chaos induces phenomena such as chaos synchronization and chaotic resonance (reviewed in^1,2,35^). In this study, we focused on chaotic resonance, which can be interpreted as the phenomenon replacing the fluctuation source in stochastic resonance by chaos instead of using additive noise^35^. Chaotic resonance can be applied in two forms. First, the signal response can be enhanced by applying an external deterministic chaotic signal instead of external stochastic noise^36–38^. Specifically, the signal generated by external chaotic systems is applied to a dynamical system with bi-stable states by inputting weak signals^36–38^. Second, the signal response can be enhanced by dynamics with intrinsic chaotic behavior instead of applying external chaotic signals produced in other systems (we considered the second form in this study)^37,39–42^. Chaotic resonance is fed to the system with chaos–chaos intermittency, where the chaotic orbit goes back and forth among separate regions^36,37,39–41,43^. In chaotic resonance, synchronization of chaos–chaos intermittency against small external signals can be induced, and its degree can be maximized close to the condition for attractor merging bifurcation (reviewed in^35^). This chaotic resonance has been found in many chaotic systems including one-dimensional cubic maps, the Chua’s circuit, Lorenz system, and Duffing oscillator^36,37,39–41^. In neural systems, the study of chaotic resonance has led to findings such as associative chaotic neural network models and a discrete neural system which is composed of excitatory and inhibitory neurons^43–46^. Moreover, recent studies of chaotic resonance have been focused on various types of neural systems, such as cerebellar learning systems and spiking neuron models with various types of spiking patterns^47–52^.

Compared to stochastic resonance, several studies have reported that the sensitivity of chaotic resonance is higher^45,46^. Still, few studies have addressed applications of chaotic resonance, possibly because in chaotic resonance, the chaotic state must be properly adjusted for obtaining resonance by the parameters of the internal system. In a large proportion of cases, particularly in biological system, adjusting internal parameters from outside cannot be realized. To solve this problem, we previously developed a method to control the chaotic state for generating chaotic resonance through an external feedback signal^53^. The feedback signal reduces the local maximum and minimum of a map function inducing chaos–chaos intermittency and separating the merged attractor. Consequently, chaotic resonance is achieved without rectifying internal parameters, in a method denominated reduced region of orbit (RRO) feedback. Although other conventional methods to control chaotic states by external signals exist, such as the Ott–Grebogi–Yorke method^54^, delayed feedback^55,56^, and *H*_∞_ control^57^, they eliminate the chaotic dynamics by stabilizing equilibrium and transitioning to a stable periodic state by applying external perturbations. In contrast, RRO feedback adjusts the chaotic state without eliminating it to generate chaotic resonance^53^.

RRO feedback has been used in a discrete cubic map, coupled cubic maps, and a discrete neural system composed of excitatory/inhibitory neurons^44^, successfully inducing chaotic resonance^53,58,59^. However, the feedback signal has been only utilized for separating the merged attractor in these studies. Therefore, if the system behaviour exhibits the condition for separating the attractor, chaotic resonance cannot be controlled because attractor merging is needed instead of separation. Further, the performance of chaotic resonance induced by feedback and that of stochastic resonance by additive noise remain to be evaluated and compared.

We hypothesized that the separated attractor can be merged by negative feedback in RRO feedback. Therefore, chaotic resonance can be induced even under a separated attractor. To prove this hypothesis, we applied negative feedback to a discrete neural system^44^ in this study. The induced chaotic resonance was evaluated regarding feedback strength, internal parameters of the neural system, and input signal amplitude/frequency. Finally, we compared the signal sensitivity of induced chaotic resonance with that of stochastic resonance by additive noise.

## Methods

### Neural system model

**Figure 1:**
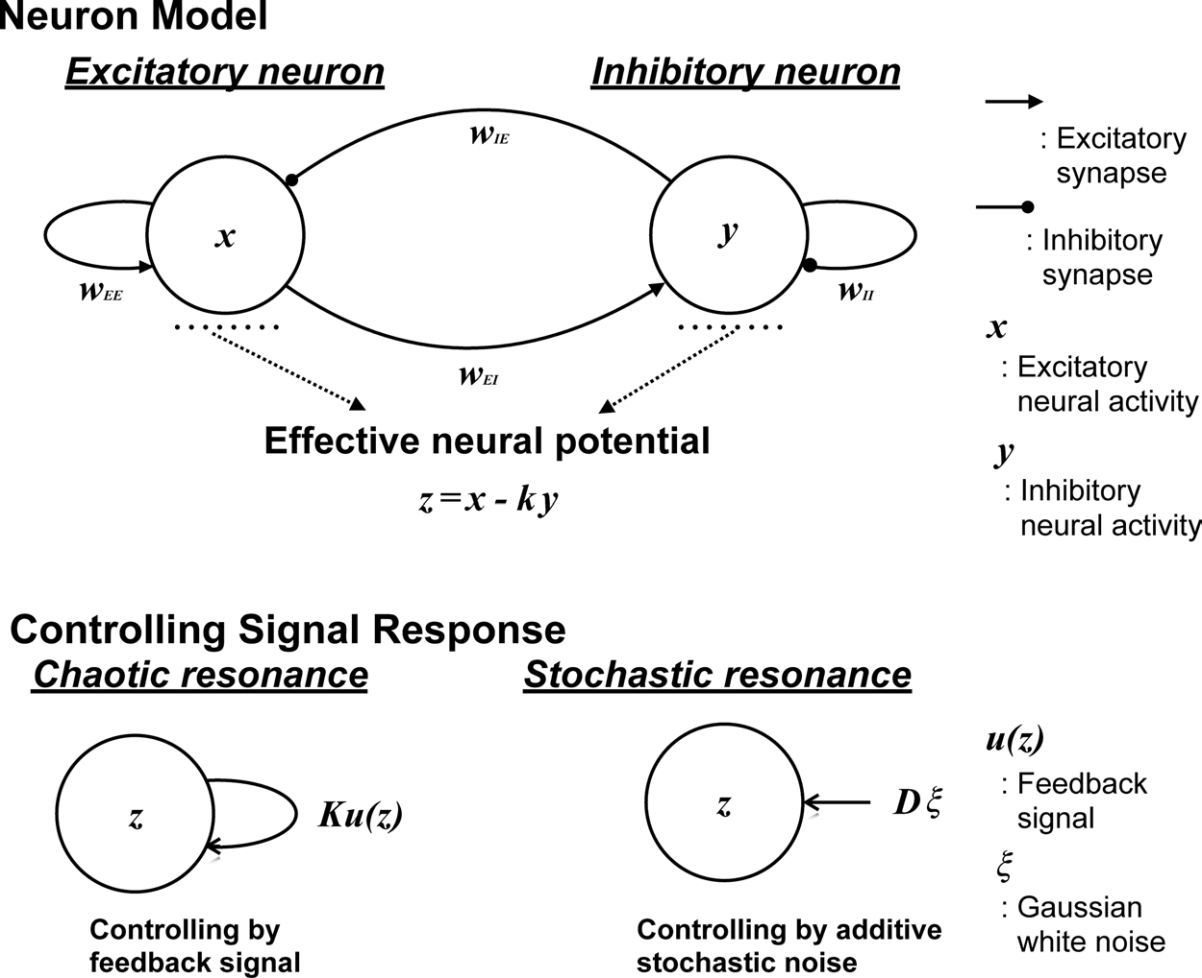
Discrete neural system composed of excitatory and inhibitory neurons^44^ (top graph). Under constraint *w*_*EI*_/*w*_*EE*_ = *w*_*II*_/*w*_*IE*_ = *k*, the two-dimensional dynamics of *x*(*t*) and *y*(*t*) reduces to effective neural potential *z*(*t*) = *x*(*t*)−*ky*(*t*). Control method for signal response using chaotic resonance by a reduced region of orbit (RRO) feedback signal *Ku*(*z*) and stochastic resonance by Gaussian white noise *Dξ* (bottom graph).

Figure 1 illustrates the discrete neural system composed of excitatory/inhibitory neurons developed by Sinha^44^ and considered in this study. The dynamics of the states for excitatory neuron *x*(*t*) and inhibitory neuron *y*(*t*) is expressed as

1 $$x(t+1)={F}_{a}({w}_{EE}x(t)-{w}_{EI}y(t)),$$

2 $$y(t+1)={F}_{b}({w}_{IE}x(t)-{w}_{II}y(t)).$$

Here, *w*_*EE*_ and *w*_*EI*_ represent excitatory synaptic weights between excitatory neurons and from excitatory to inhibitory neurons, respectively, *w*_*II*_ and *w*_*IE*_ represent inhibitory synaptic weights between inhibitory neurons and from inhibitory to excitatory neurons, respectively. Activation functions *F*_*a*_ and *F*_*b*_ are given by *F*_*a*_(*X*) = −1 for *X* < −1/*a*, *F*_*a*_(*X*) = *aX* for −1/*a* ≤ *X* ≤ 1/*a*, *F*_*a*_(*X*) = 1 for *X* > 1/*a*, *F*_*b*_(*Y*) = −1 for *Y* < −1/*b*, *F*_*b*_(*Y*) = *bY* for −1/*b* ≤ *Y* ≤ 1/*b*, and *F*_*b*_(*Y*) = 1 for *Y* > 1/*b*. Parameters 1/*a* and 1/*b* correspond to the activation threshold for the states of excitatory and inhibitory neurons, respectively. Under constraint *w*_*EI*_/*w*_*EE*_ = *w*_*II*_/*w*_*IE*_ = *k*, the two-dimensional dynamics of *x*(*t*) and *y*(*t*) reduces to effective neural potential *z*(*t*) = *x*(*t*)−*ky*(*t*), whereas the one-dimensional dynamics is given by

3 $$z(t+1)=F(z(t))={F}_{a}(z(t))-k{F}_{b}(z(t)).$$

To control the signal response in the neural system, we use chaotic resonance or stochastic resonance (Fig. 1). In both control approaches, the signal response is controlled by attractor merging (chaos–chaos intermittency). In chaotic resonance, attractor merging is controlled by an external feedback signal, whereas in stochastic resonance, attractor merging is controlled by additive noise. Specifically, to induce chaotic resonance by controlling the chaos–chaos intermittency of effective neural potential *z*(*t*), we applied an RRO feedback term *u*(*z*)^53^ as follows:

4 $$z(t+1)=F(z(t))+Ku(z(t)),$$

5 $$u(z)=-(z-{z}_{d})\exp (-{(z-{z}_{d})}^{2}/(2{\sigma }^{2})).$$

Here, *K*, *z*_*d*_, and *σ* are the RRO feedback strength, merging point of each chaotic attractors, and a parameter to determine the region for RRO feedback effect, respectively.

In this simulation, we used set parameters to *a* = 5.95, 5.96, 5.97, *b* = 3.42, and *k* = 1.3811^44^. Consequently, the orbit of *z*(*t*) is trapped to its positive or negative regions. Based on our previous study^53^, *z*_*d*_ and *σ* were set to *z*_*d*_ = 0 for the divided points of each chaotic region *z* = 0, and the distance from this divided point *z*(0) to the local maximum/minimum $\sigma =\frac{1}{a}$. In addition, the positive region of *K* was investigated, where RRO feedback signal *Ku*(*z*) suppresses chaos–chaos intermittency and separates the merged chaotic attractor^59^. In contrast, we focused on the negative region in the present study. In the negative region, we assumed that RRO feedback signal *Ku*(*z*) enhances chaos–chaos intermittency and merges the separated chaotic attractor.

**Figure 2:**
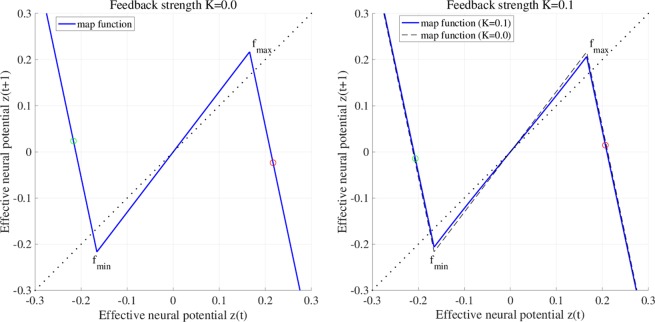
Map function *F*(*z*) + *K*(*u*(*z*)) according to external feedback signals with positive strength for *a* = 6.03. The left and right graphs indicate map function with *K* = 0.0 satisfying attractor merging conditions and that with *K* = 0.1 not satisfying attractor merging conditions. The red and green circles indicate *F*(*f*_max_) + *K*(*u*(*f*_max_)) and *F*(*f*_min_) + *K*(*u*(*f*_min_)), respectively. RRO feedback separates the merged attractor due to decreasing absolute values of *f*_max_ and *f*_min_.

**Figure 3:**
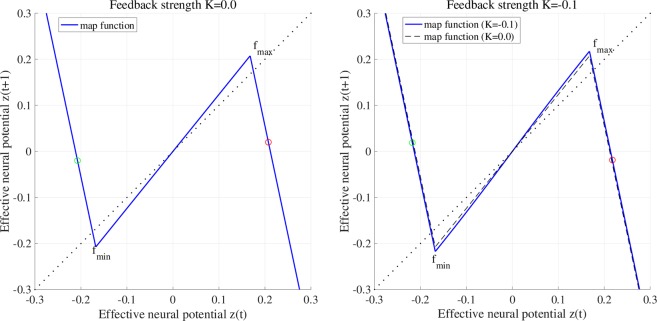
Map function *F*(*z*) + *K*(*u*(*z*)) according to external feedback signals with negative feedback strength for *a* = 5.96. The left and right graphs indicate map function with *K* = 0.0 not satisfying attractor merging conditions and that with *K* = −0.1 satisfying attractor merging conditions. RRO feedback allows to control the merging attractor conditions due to increasing absolute values of *f*_max_ and *f*_min_.

**Figure 4:**
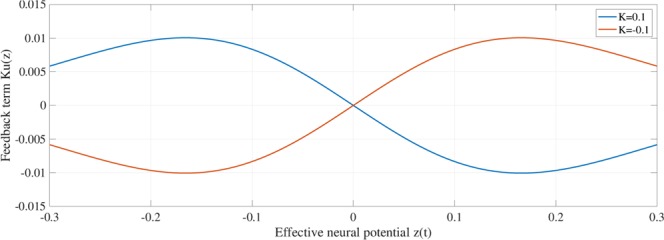
Feedback signal *K*(*u*(*z*)) for *K* = 0.1, −0.1. For positive feedback strength (*K* = 0.1), local maximum and minimum of *K*(*u*(*z*)) are located at the local minimum and maximum of the map function of *F*, respectively. For negative feedback strength (*K* = −0.1), this location is the opposite.

To explain the effect of RRO feedback signal *Ku*(*z*), Figs 2 and 3 show map function of *F*(*z*) + *K*(*u*(*z*)) according to external feedback signals. Attractor merging (chaos–chaos intermittency) occurs if *F*(*f*_max_) + *K*(*u*(*f*_max_)) < 0 and *F*(*f*_min_) + *K*(*u*(*f*_min_)) > 0, where *f*_max_ and *f*_min_ are the local maximum and minimum of the map function. For internal neural parameter *a* = 6.03 and feedback strength *K* = 0, the attractor merging conditions are satisfied (left graph in Fig. 2). Applying positive feedback (*K* = 0.1, Fig. 4), the absolute values of *f*_max_ and *f*_min_ are reduced, and the attractor merging conditions are not satisfied, as shown in the right graph of Fig. 2. In our previous work^59^, we controlled chaotic resonance using this suppressing effect. For *a* = 5.96 and *K* = 0, the attractor merging conditions are not satisfied (left graph in Fig. 3). However, applying negative feedback signal *Ku*(*z*) (*K* = −0.1, Fig. 4), the absolute values of *f*_max_ and *f*_min_ increase, and the attractor merging conditions are satisfied, as shown in the right graph of Fig. 3.

To evaluate the signal response during chaotic resonance, an external sinusoidal signal *S*(*t*) = *A*sin2*π*Ω*t* can be applied:

6 $$z(t+1)=F(z(t))+Ku(z(t))+S(t).$$

For stochastic resonance, additive white Gaussian noise ξ(*t*) with zero mean and unit variance can be applied to Eq. (6) as follows:

7 $$z(t+1)=F(z(t))+Ku(z(t))+S(t)+D\xi (t).$$

Here, *D* represents the noise strength.

### Indices for signal response, chaotic state, and controlling attractor merging

We consider the synchronization between sign change of effective neural potential *z*(*t*) and input signal *S*(*t*) as signal response by utilizing the correlation coefficient between binarized time series *z*(*t*) being *Z*(*t*) (*Z*(*t*) = 1 if *z*(*t*) ≥ 0; *Z*(*t*) = −1 otherwise) and the time series of input signal *S*(*t*):

8 $$C(\tau )=\frac{{C}_{SZ}(\tau )}{\sqrt{{C}_{SZ}{C}_{ZZ}}},$$

9 $${C}_{SZ}(\tau )=\langle (S(t+\tau )-\langle S\rangle )(Z(t)-\langle Z\rangle )\rangle ,$$

10 $${C}_{SS}=\langle {(S(t)-\langle S\rangle )}^{2}\rangle ,$$

11 $${C}_{ZZ}=\langle {(Z(t)-\langle Z\rangle )}^{2}\rangle ,$$

where 〈⋅〉 denotes averaging over *t* and *τ* is the time delay.

To evaluate the instability of the *z*(*t*) orbit as chaos index, the Lyapunov exponent is employed^60^:

12 $$\lambda =\frac{1}{\tau M}\mathop{\sum }\limits_{k=1}^{M}\,\mathrm{ln}(\frac{{d}^{k}({t}_{l}=\tau )}{{d}^{k}({t}_{l}=0)}),$$

where *d*^*k*^(*t*_*l*_ = 0) = *d*_0_ (*k* = 1, 2, …, *M*) indicates *M* perturbed initial conditions at *t* = *t*_0_ + (*k*−1)*τ* adding to *z*(*t*), whose temporal evolution for *t*_*l*_∈[0:*τ*] is ${d}^{k}({t}_{l}=\tau )=(z(t)-z^{\prime} (t)){|}_{t={t}_{0}+k\tau }$, with *z*′(*t*) denoting a perturbed orbit.

Finally, to confirm the frequency control effect of chaos–chaos intermittency by external RRO feedback signal, we use the occurrence probability of chaos–chaos intermittency:

13 $${P}_{t}=\frac{{f}_{{\rm{cc}}}}{T},$$

where *T* and *f*_cc_ denote the number of iterations and frequency of chaos–chaos intermittency, respectively.

## Results

### Attractor merging induced by internal neural parameters

**Figure 5:**
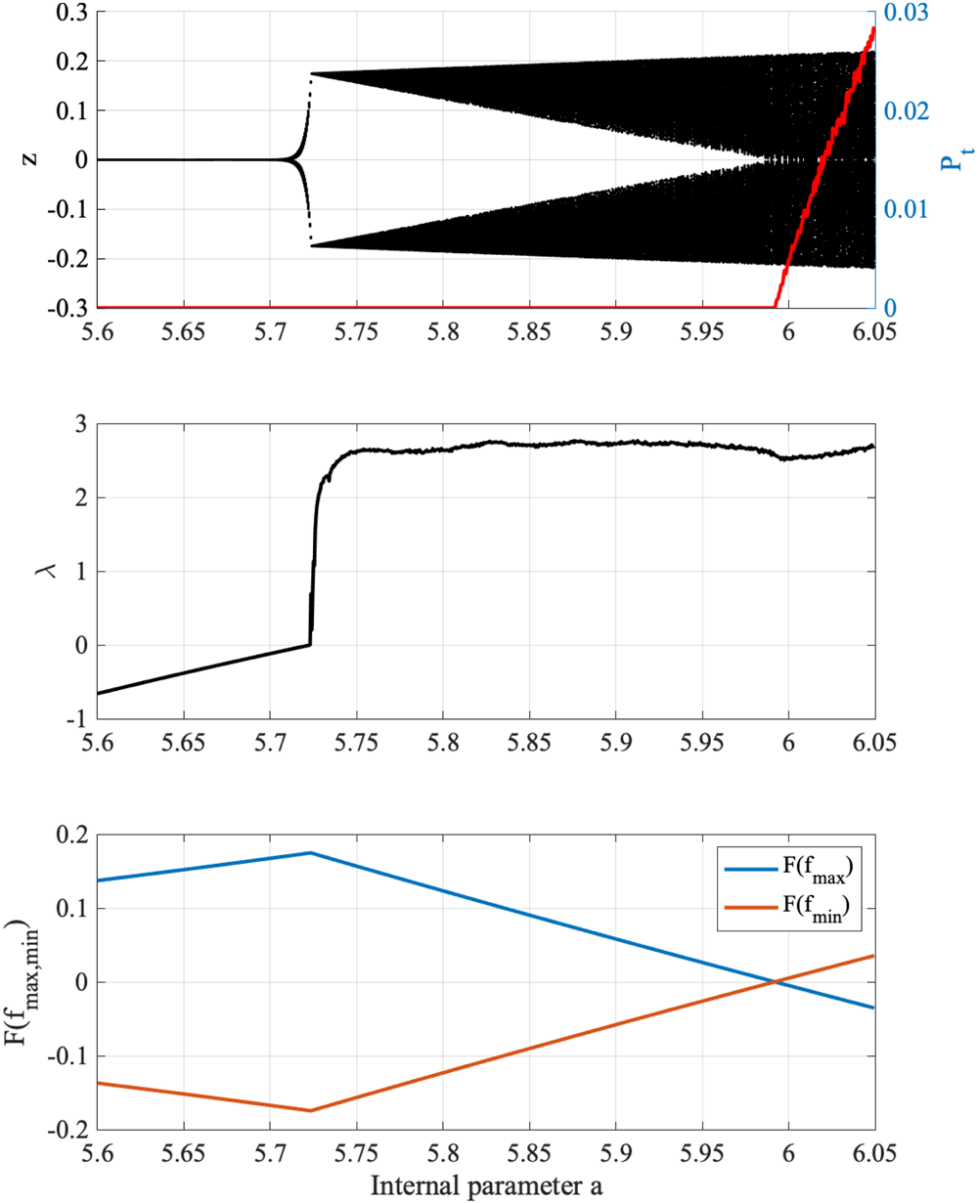
Chaotic attractor merging by internal neural parameter *a*. As function of parameter *a*, bifurcation diagram of effective neural potential *z*(*t*) (black dot) and occurrence probability of chaos–chaos intermittency *P*_*t*_ (red line) (top) are shown using negative and positive initial values of *z*(0). Lyapunov exponent *λ* (middle) and *F*(*f*_max, min_) (bottom). The chaotic attractor (*λ* > 0) is merged when satisfying condition for attractor merging *F*(*f*_max_) < 0, *F*(*f*_min_) > 0 for *a* $\gtrsim$ 5.99. All graphs are plotted according to internal neural parameter *a*.

We demonstrate the dependence of dynamics on internal neural parameter *a*. Figure 5 shows the effective neural potential *z*(*t*) bifurcation diagram, occurrence probability of chaos–chaos intermittency *P*_*t*_, Lyapunov exponent *λ* and *F*(*f*_max_), *F*(*f*_min_) according to internal neural parameter *a*. In the bifurcation diagram, two kinds of initial values for *z*(0) (i.e. negative and positive values) are used. The chaotic attractor (*λ* > 0), which is divided into positive and negative regions in $5.72\lesssim a\lesssim 5.99$, is merged for $a\gtrsim 5.99$ when satisfying attractor merging condition *F*(*f*_max_) < 0, *F*(*f*_min_) > 0.

### Control of attractor merging

**Figure 6:**
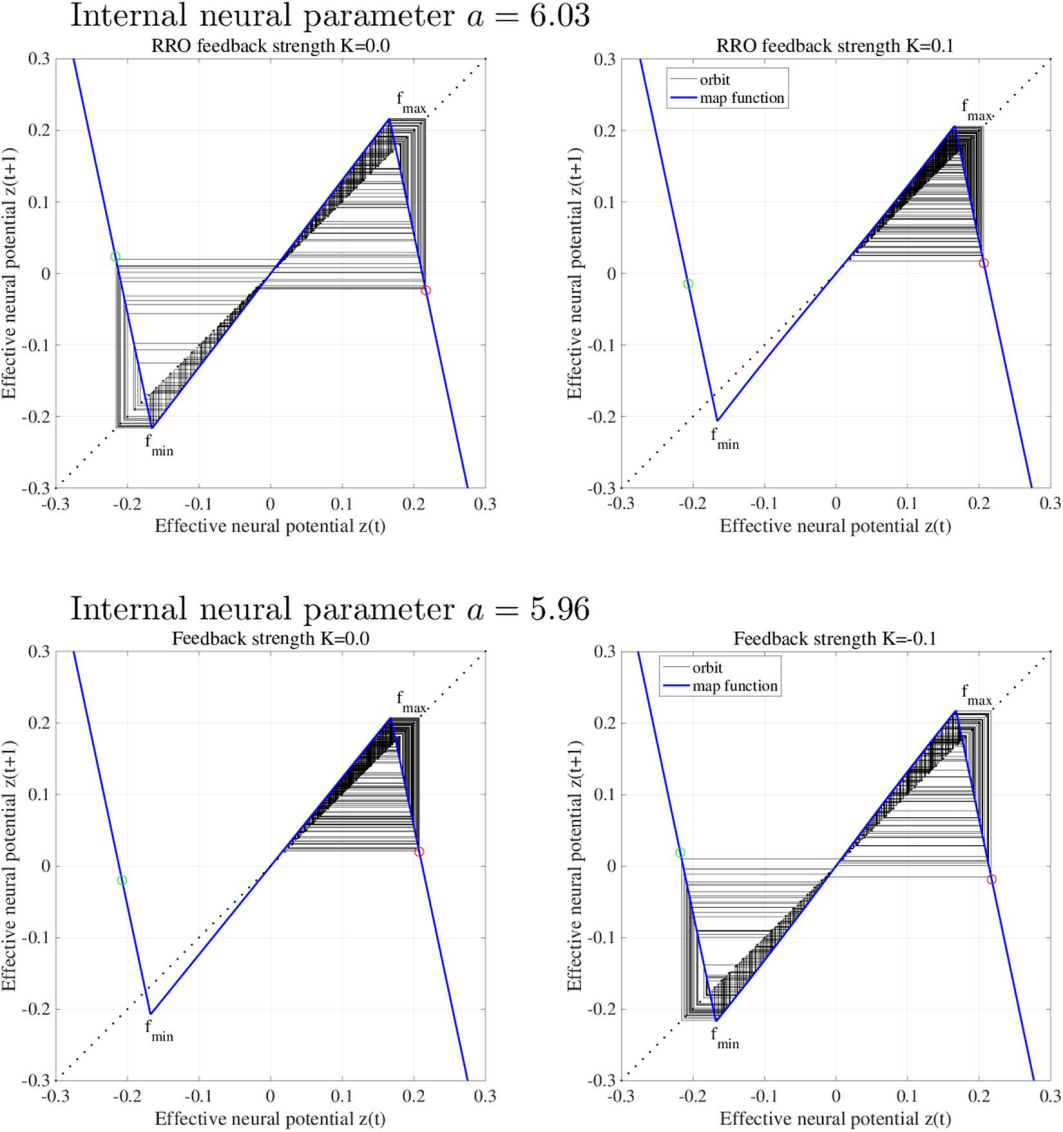
Effect of RRO feedback for separating the merged attractor (top) and merging the separated attractor (bottom). The graphs show the *z*(*t*) map function in the neural system (blue solid line) and its orbit (black solid line) for *a* = 6.03, 5.96 without RRO feedback signal (*K* = 0) and with RRO feedback signal (*K* = 0.1,−0.1). The red and green circles indicate *F*(*f*_max_) + *K*(*u*(*f*_max_)) and *F*(*f*_min_) + *K*(*u*(*f*_min_)), respectively. *f*_max_ (*f*_min_) is local maximum (minimum) for the map function. By reducing and increasing the absolute values *f*_max_ and *f*_min_, the effects for separating and merging the attractor are induced, respectively.

**Figure 7:**
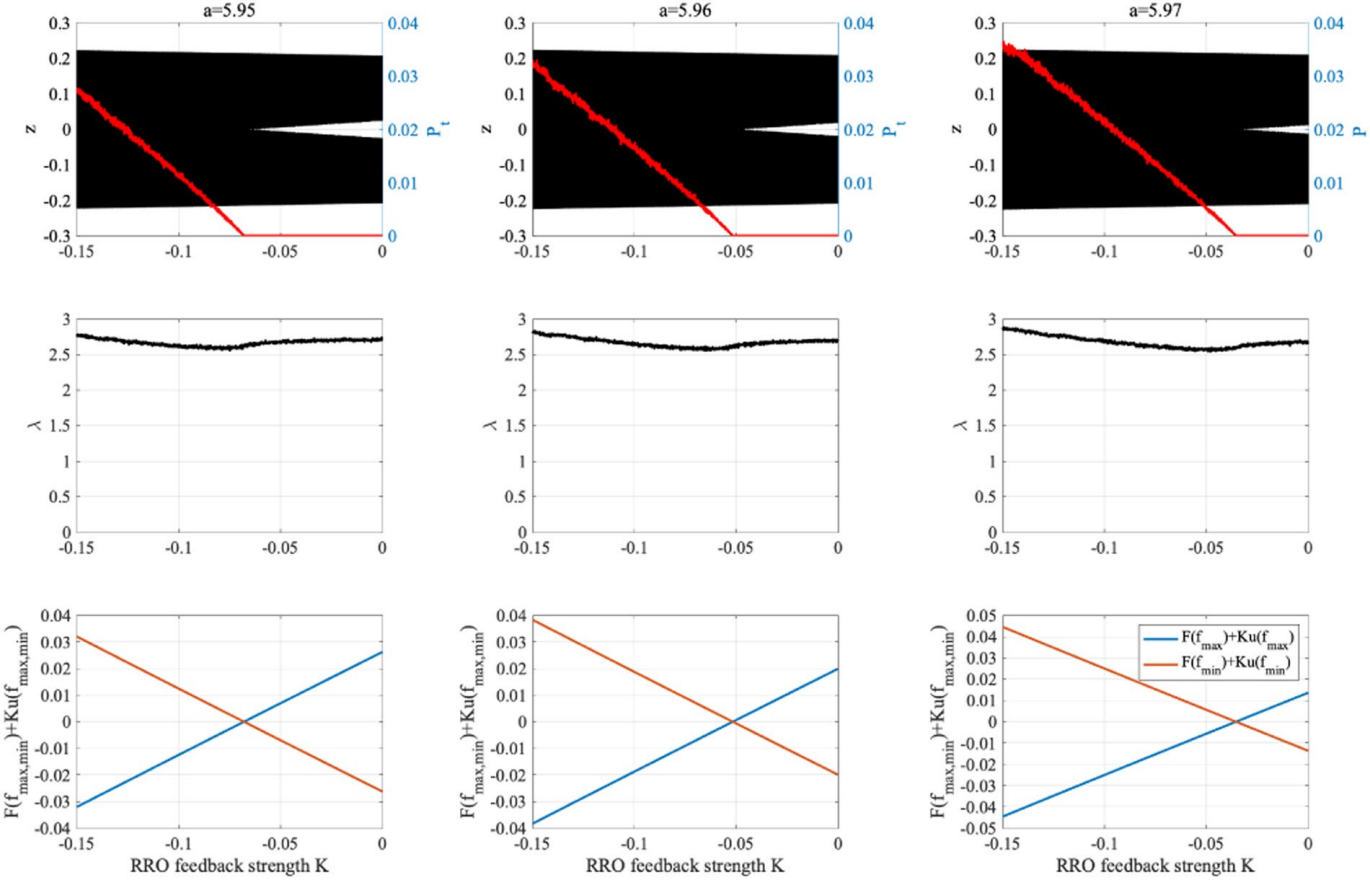
System behaviour according to RRO feedback strength *K*. As function of *K*, bifurcation diagram of *z*(*t*) (black) and occurrence probability of chaos–chaos intermittency *P*_*t*_ (red line) are shown using RRO feedback (top). Two kinds of initial values of *z*(0) (i.e. negative and positive values) are used. Lyapunov exponent *λ* (middle) and *F*(*f*_max_) + *K*(*u*(*f*_max_)), *F*(*f*_min_) + *K*(*u*(*f*_min_)) (bottom). All graphs are plotted according to feedback strength *K*. The chaotic attractor (*λ* > 0) is merged as it satisfies condition for attractor merging *F*(*f*_max_) + *K*(*u*(*f*_max_)) < 0, *F*(*f*_min_) + *K*(*u*(*f*_min_)) > 0 at $K\lesssim -\,0.068,-\,0.051,-\,0.035$ for *a* = 5.95, 5.96, 5.97, respectively. Decreasing internal neural parameter *a* from the attractor merging, *a* ≈ 5.99, shown in Fig. 5, demands a larger absolute value of negative feedback strength *K*.

Consider the effect of RRO feedback signal for separating and merging the attractor. Figure 6 shows the *z*(*t*) map function and its orbit for *a* = 6.03 without RRO feedback signal (*K* = 0) and with positive RRO feedback signal (*K* = 0.1). Without RRO feedback, attractor merging condition *F*(*f*_max_) < 0, *F*(*f*_min_) > 0 is satisfied, and therefore *z*(*t*) exhibits chaos–chaos intermittency between the negative and positive *z*(*t*) regions. With RRO feedback signal, this condition is not satisfied, and the merged attractor is separated, while *z*(*t*) is trapped in the negative or positive region, depending on the *z*(*t*) initial value. Figure 6 also shows the *z*(*t*) map function and its orbit for *a* = 5.96 without RRO feedback signal (*K* = 0) and with negative RRO feedback signal (*K* = −0.1). Without feedback, the condition for attractor merging, *F*(*f*_max_) < 0, *F*(*f*_min_) > 0, is not satisfied, and therefore *z*(*t*) is trapped in the negative or positive region. Applying negative feedback (*K* = −0.1), the merging condition is satisfied, and thus *z*(*t*) exhibits chaos–chaos intermittency. To evaluate the dependence of the system behaviour on negative RRO feedback strength *K* in more detail, Fig. 7 depicts the *z*(*t*) bifurcation diagram, *P*_*t*_, *λ*, *F*(*f*_max_) + *K*(*u*(*f*_max_)), and *F*(*f*_min_) + *K*(*u*(*f*_min_)) as function of *K* for *a* = 5.95, 5.96, 5.97. The separated chaotic attractor (*λ* > 0) merges as it satisfies the condition for attractor merging, *F*(*f*_max_) + *K*(*u*(*f*_max_)) < 0, *F*(*f*_min_) + *K*(*u*(*f*_min_)) > 0 and *P*_*t*_ > 0 in $K\lesssim -\,0.068,-\,0.051,-\,0.035$ for *a* = 5.95, 5.96, 5.97, respectively. Decreasing internal neural parameter *a* from the attractor merging, *a* ≈ 5.99, demands a larger absolute value of negative feedback strength *K*.

**Figure 8:**
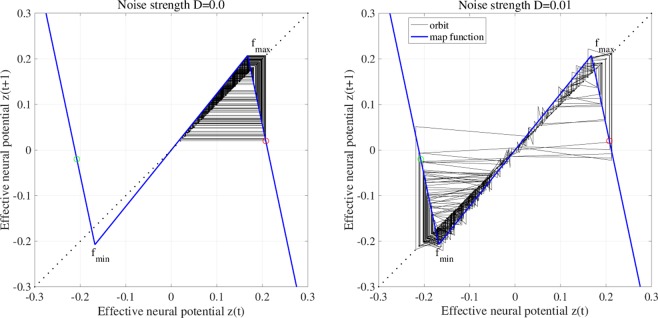
Effect of adding noise to merge separated attractor without RRO feedback (*K* = 0). The graphs show the *z*(*t*) map function (blue solid line) and its orbit (black solid line) for *a* = 5.96 without noise (*D* = 0) and with noise (*D* = 0.01). The red and green circles indicate *F*(*f*_max_) and *F*(*f*_min_), respectively. Despite of attractor merging condition (*F*(*f*_max_) < 0, *F*(*f*_min_) > 0) is not satisfied by the effect of additive noise, and attractor merging is induced.

**Figure 9:**
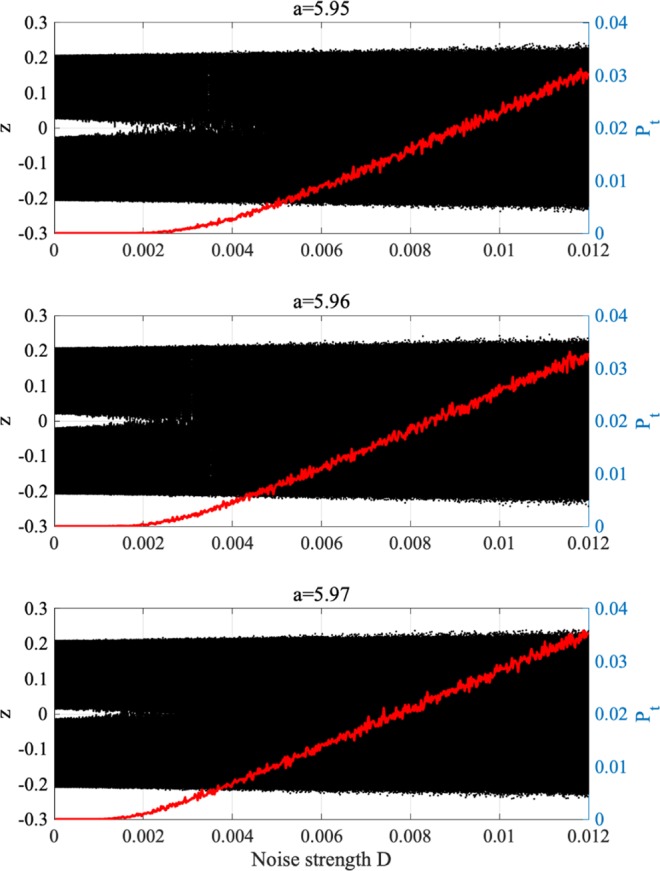
System behaviour according to noise strength *D*. Bifurcation diagram of *z*(*t*) (black) and occurrence probability of chaos–chaos intermittency *P*_*t*_ (red line). Two kinds of initial values of *z*(0) (i.e. negative and positive) are used. All graphs are plotted according to noise strength *D*. The chaotic attractor is merged for *D* $\gtrsim$ 2.5 × 10^−3^, 2.0 × 10^−3^, 1.5 × 10^−3^ at *a* = 5.95, 5.96, 5.97, respectively. Decreasing internal neural parameter *a* from the attractor merging, *a* ≈ 5.99, indicated in Fig. 5, demands a larger noise strength *D* for attractor merging.

Next, we evaluate the effect of additive noise for merging the attractor. Figure 8 shows the map function of *z*(*t*) and its orbit for *a* = 5.96 without noise (*D* = 0). The attractor is separating (*F*(*f*_max_) > 0, *F*(*f*_min_) < 0). Adding noise (*D* = 0.01), the attractor is merged, and *z*(*t*) exhibits chaos–chaos intermittency. The dependence of *z*(*t*) behaviour on noise strength *D* is investigated in detail. Figure 9 shows the bifurcation diagram of *z*(*t*) and *P*_*t*_ according to *D* for *a* = 5.95, 5.96, 5.97. If $D\gtrsim 2.5\times {10}^{-3}$, 2.0 × 10^−3^, 1.5 × 10^−3^ in *a* = 5.95, 5.96, 5.97, respectively, the attractor is merged for *P*_*t*_ > 0. Decreasing internal neural parameter *a* from the attractor merging, *a* ≈ 5.99, shown in Fig. 5, demands a larger noise strength *D*.

### Control signal response in chaotic resonance

**Figure 10:**
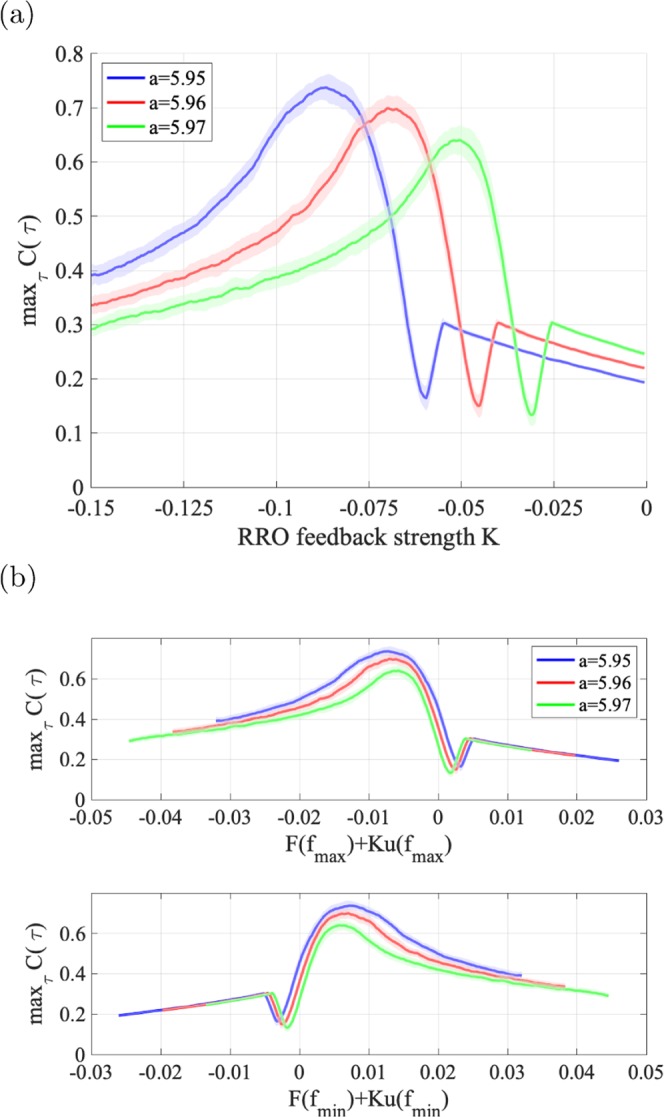
Signal response according to feedback strength *K* under chaotic resonance. (**a**) Correlation coefficient max_*τ*_ *C*(*τ*) between input signal *S*(*t*) and binarized *z*(*t*) according to feedback strength *K*. (**b**) Correlation max_*τ*_ *C*(*τ*) (corresponding to values in Fig. 10(a)) according to *F*(*f*_max_) + *K*(*u*(*f*_max_)) and *F*(*f*_min_) + *K*(*u*(*f*_min_)) (corresponding to values in Fig. 7) for −0.15 ≤ *K* ≤ 0.0. Solid lines and shaded areas represent the mean and standard deviation of max_*τ*_ *C*(*τ*) among 10 trials, respectively. For every value of *a*, correlation coefficient max_*τ*_ *C*(*τ*) exhibits a unimodal peak at the slightly merged settings for feedback strength (i.e. chaotic resonance occurs).

**Figure 11:**
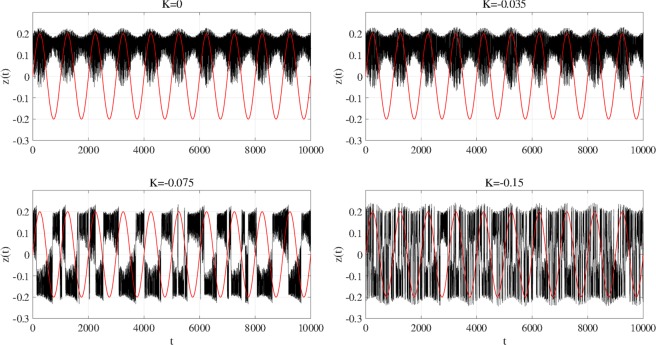
Time series of *z*(*t*) (black line) and input sinusoidal signal *S*(*t*) (red line) for internal neural parameter *a* = 5.96 (corresponding to Fig. 10). Without RRO feedback signal (*K* = 0) and with weak RRO feedback strength (*K* = −0.035), attractor switching occurs around the peaks of input signal *S*(*t*). However, switching is not maintained for more than one iteration. In contrast, at appropriate feedback strength (*K* = −0.075), where *K* corresponds to the peak of max_*τ*_ *C*(*τ*) (*K* = −0.075), the attractor switches at the period of *S*(*t*), and thus chaos–chaos intermittency synchronizes with the input signal. At even stronger negative *K* (*K* = −0.15), the very high chaos–chaos intermittency frequency does not allow to confirm its synchronization with input signal *S*(*t*).

We first evaluate the signal response according to feedback strength *K* under chaotic resonance. Figure 10 shows correlation coefficient max_*τ*_ *C*(*τ*) between input sinusoidal signal *S*(*t*) and binarized *z*(*t*) according to RRO feedback strength *K*, *F*(*f*_max_) + *K*(*u*(*f*_max_)), and *F*(*f*_min_) + *K*(*u*(*f*_min_)). Correlation coefficient max_*τ*_ *C*(*τ*) exhibits a unimodal peak at the slightly merged settings for feedback strength. To observe the system behaviour in more detail, Fig. 11 shows time series *z*(*t*) for *a* = 5.96. Without RRO feedback signal (*K* = 0), attractor switching occurs around the peaks of input signal *S*(*t*). However, switching is not maintained for more than one iteration. Stronger feedback (*K* = −0.035) increases the switching frequency, but again, switching is not maintained for more than one iteration. In contrast, at appropriate feedback strength, where *K* corresponds to the peak of max_*τ*_ *C*(*τ*) (*K* = −0.075), the attractor switches at the period of *S*(*t*), and thus chaos–chaos intermittency synchronizes with the input signal. At even stronger negative *K* (*K* = −0.15), the very high chaos–chaos intermittency frequency does not allow to confirm its synchronization with input signal *S*(*t*).

**Figure 12:**
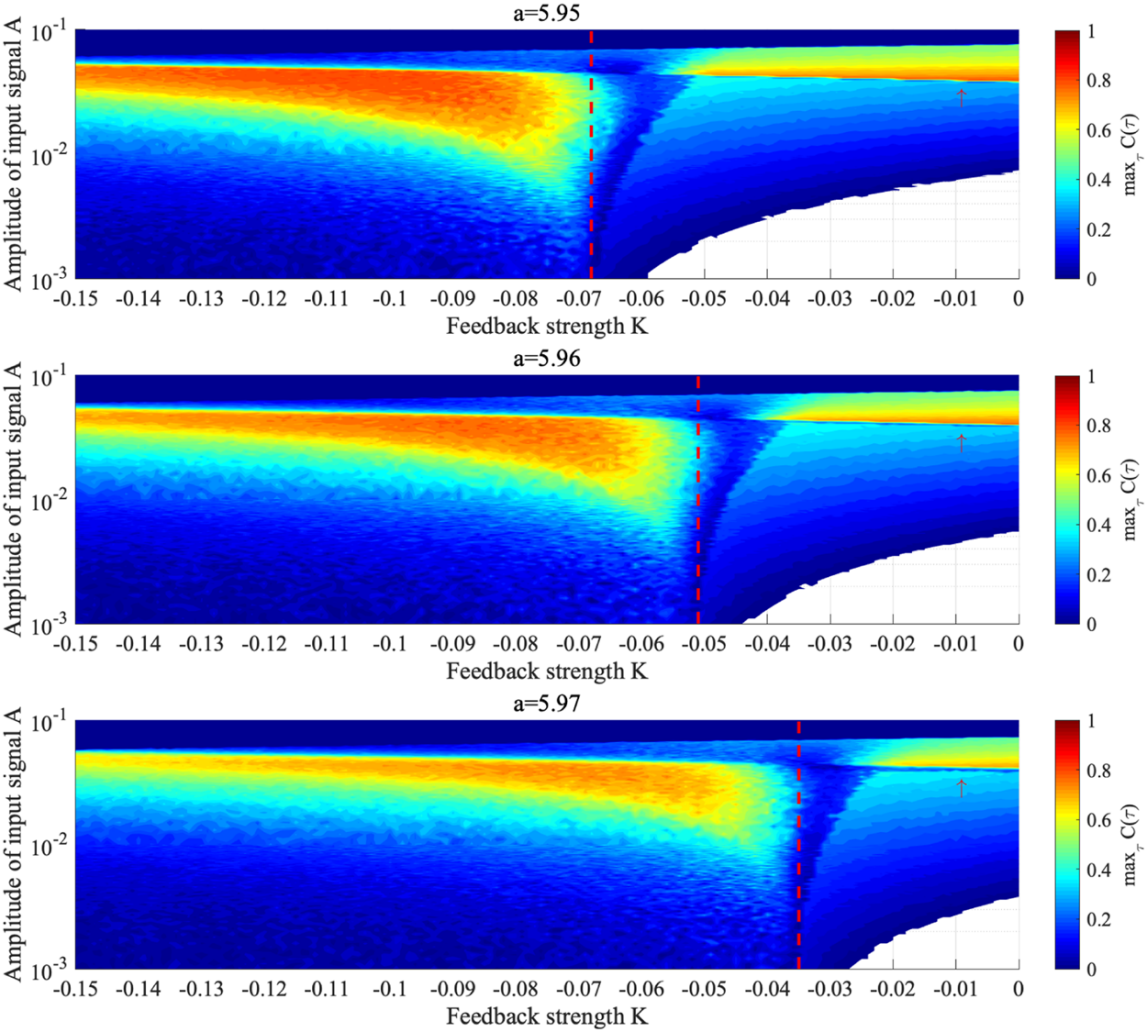
Correlation coefficient max_*τ*_ *C*(*τ*) between input sinusoidal signal *S*(*t*) and binarized *z*(*t*) according to feedback strength *K* and signal amplitude *A* under chaotic resonance. In the white region, the behaviour of *z*(*t*) indicates the absence of chaos–chaos intermittency. The red arrow indicates the region where synchronization occurs without feedback. The smaller region of *K*, delimited by the red dashed line, indicates attractor merging without input sinusoidal signal *S*(*t*). In the region of smaller *K* values where attractor merging induced and $2.0\times {10}^{-3}\lesssim A\lesssim 6.0\times {10}^{-2}$, chaotic resonance occurs for every value of internal neural parameter *a*. Around the values of *K* indicated as red dashed lines, the sensitivity of chaotic resonance becomes high. Consequently, the signal response of chaotic resonance is maximized at appropriate input signal amplitude.

Then, we evaluate the sensitivity of the signal response in chaotic resonance. Figure 12 shows correlation coefficient max_*τ*_ *C*(*τ*) between input sinusoidal signal *S*(*t*) and binarized *z*(*t*) according to feedback strength *K* and signal amplitude *A* under chaotic resonance. In amplitude region $2.0\times {10}^{-3}\lesssim A\lesssim 6.0\times {10}^{-2}$ and for *K* ≈ −0.068 (*a* = 5.95), −0.051 (*a* = 5.96), −0.035 (*a* = 5.97), where attractor merging occurs without input sinusoidal signal *S*(*t*), high values of max_*τ*_ *C*(*τ*) $\gtrsim$ 0.3 are confirmed for every value of *a* (i.e. chaotic resonance occurs).

**Figure 13:**
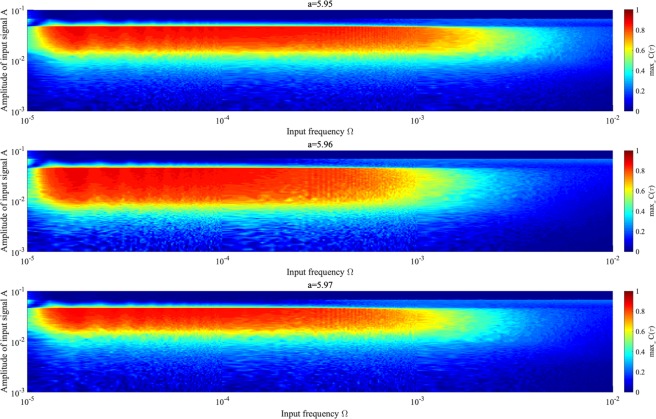
Correlation coefficient max_*τ*_ *C*(*τ*) between input sinusoidal signal *S*(*t*) and binarized *z*(*t*) according to signal frequency Ω under chaotic resonance. *K* = −0.09, −0.06, −0.05 is considered for *a* = 5.95, 5.96, 5.97, respectively, corresponding to *K* values where peak max_*τ*_ *C*(*τ*) occurs in Fig. 10. Correlation coefficient max_*τ*_ *C*(*τ*) exhibits high values for $2.0\times {10}^{-5}\lesssim {\rm{\Omega }}\lesssim 1.0\times {10}^{-3}$ and weak input signal $2.0\times {10}^{-3}\lesssim A\lesssim 6.0\times {10}^{-2}$ for every value of *a*. Consequently, the signal response of chaotic resonance is maximized at appropriate input signal frequency.

To investigate the relationship between input signal amplitude and signal response frequency, Fig. 13 shows correlation max_*τ*_ *C*(*τ*) according to the amplitude and frequency of *S*(*t*) for various values of *K* and *a*, where *K* corresponds to peaks in correlation max_*τ*_ *C*(*τ*) (Fig. 10). High values of max_*τ*_ *C*(*τ*) $\gtrsim$ 0.3 are achieved in region $2.0\times {10}^{-5}\lesssim {\rm{\Omega }}\lesssim 1.0\times {10}^{-3}$ and $2.0\times {10}^{-3}\lesssim A\lesssim 6.0\times {10}^{-2}$.

### Control of signal response in stochastic resonance

**Figure 14:**
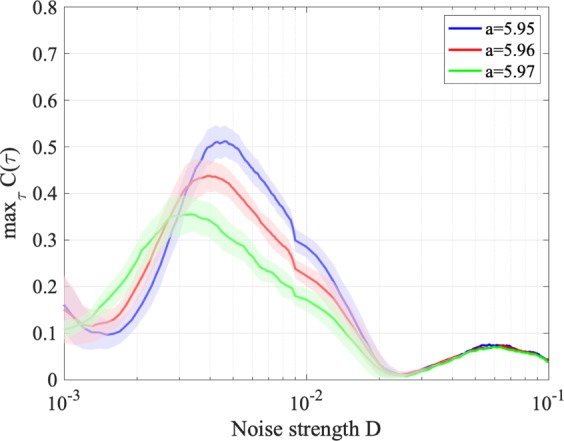
Correlation coefficient max_*τ*_ *C*(*τ*) between input sinusoidal signal *S*(*t*) and binarized *z*(*t*) according to noise strength *D* under stochastic resonance. Solid lines and shaded areas indicate the mean and standard deviation of max_*τ*_ *C*(*τ*) among 10 trials, respectively. Correlation coefficient max_*τ*_ *C*(*τ*) exhibits a unimodal peak around the slightly merged settings for noise strength given in Fig. 9. Therefore, stochastic resonance is induced.

**Figure 15:**
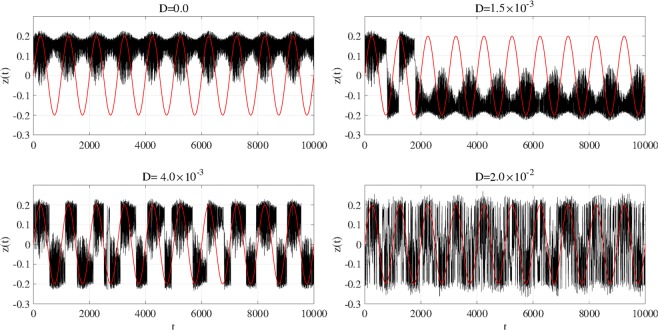
Time series of *z*(*t*) (black line) and input sinusoidal signal *S*(*t*) (red line) for internal neural parameter *a* = 5.96 (corresponding to Fig. 14). Without additive noise (*D* = 0) and with weak noise strength (*D* = 1.5 × 10^−3^), attractor switching occurs around the peaks of input signal *S*(*t*). However, switching is almost lost in more than one iteration. For stronger noise, which corresponds to the value of *D* at which the peak of max_*τ*_ *C*(*τ*) occurs (*D* = 4.0 × 10^−3^), the attractor switches at the period of *S*(*t*), and chaos–chaos intermittency synchronizes with the input signal. At even stronger *D* (*D* = 2.0 × 10^−2^), the very high frequency of chaos–chaos intermittency does not allow to confirm its synchronization with input signal *S*(*t*).

By controlling attractor merging using additive noise, stochastic resonance can be evaluated without feedback strength (*K* = 0). Figure 14 shows correlation coefficient max_*τ*_ *C*(*τ*) between input sinusoidal signal *S*(*t*) and binarized *z*(*t*) according to noise strength *D*. A single peak is confirmed around the slightly merged settings for noise strength *D* ≈ 5.0 × 10^−3^, 4.0 × 10^−3^, 3.0 × 10^−3^ at *a* = 5.95, 5.96, 5.97 respectively, given in Fig. 9. Therefore, stochastic resonance is induced. Figure 15 shows time series *z*(*t*) for *a* = 5.96 (corresponding to Fig. 14). Without additive noise (*D* = 0) and with weak noise strength (*D* = 1.5 × 10^−3^), attractor switching occurs around the peaks of input signal *S*(*t*). However, switching is almost lost in more than one iteration. For stronger noise corresponding to *D* where the peak of max_*τ*_ *C*(*τ*) occurs (*D* = 4.0 × 10^−3^), the attractor switches at the period of *S*(*t*), and chaos–chaos intermittency synchronizes with the input signal. At even stronger *D* (*D* = 2.0 × 10^−2^), the very high frequency of chaos–chaos intermittency does not allow to confirm its synchronization with input signal *S*(*t*).

**Figure 16:**
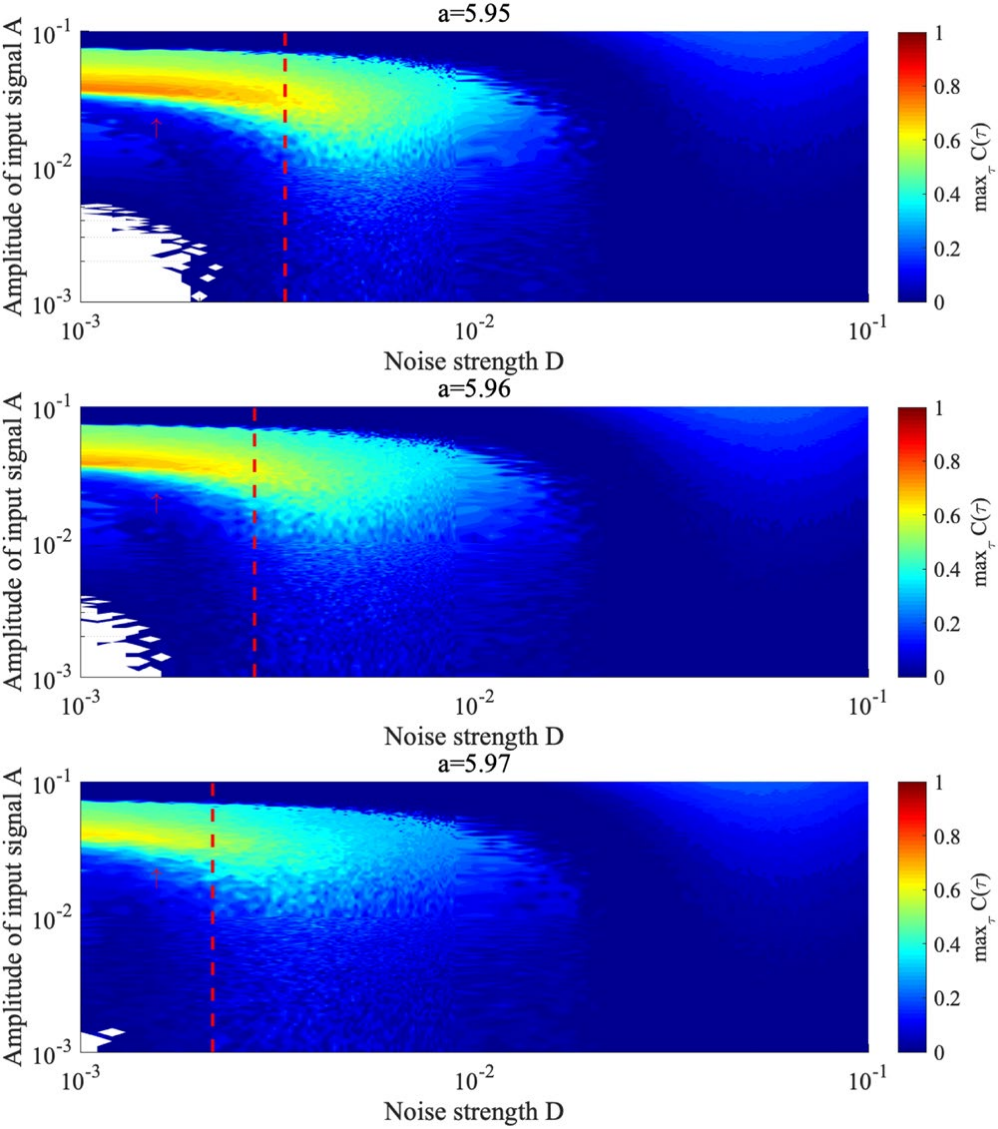
Correlation coefficient max_*τ*_ *C*(*τ*) between input sinusoidal signal *S*(*t*) and binarized *z*(*t*) according to noise strength *D* and signal amplitude *A* under stochastic resonance. In the white region, the behaviour of *z*(*t*) indicates the absence of chaos–chaos intermittency. The red arrow indicates the region where synchronization occurs without noise. The larger region of *D*, delimited by the red dashed line, indicates attractor merging without input sinusoidal signal *S*(*t*). In the region of larger *D* values from the attractor merging point (red dashed line) and $2.0\times {10}^{-2}\lesssim A\lesssim 8.0\times {10}^{-2}$, stochastic resonance occurs for every value of *a*. Compared to the chaotic resonance shown in Fig. 12, the sensitivity and degree of the signal response are lower.

Then, to evaluate the sensitivity of the signal response under stochastic resonance, Fig. 16 shows correlation coefficient max_*τ*_ *C*(*τ*) according to noise strength *D* and signal amplitude *A*. In amplitude region $2.0\times {10}^{-2}\lesssim A\lesssim 8.0\times {10}^{-2}$ and for large *D*, where attractor merging occurs without input sinusoidal signal *S*(*t*), high values of max_*τ*_ *C*(*τ*) $\gtrsim$ 0.3 are confirmed for every value of *a* (i.e. stochastic resonance occurs).

**Figure 17:**
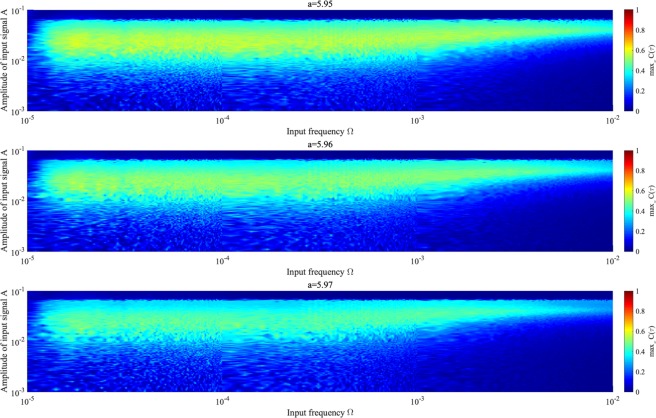
Correlation coefficient max_*τ*_ *C*(*τ*) between input signal *S*(*t*) and binarized *z*(*t*) according to signal frequency Ω and signal amplitude *A* under stochastic resonance, where *D* = 5 × 10^−3^, 4 × 10^−3^, 3 × 10^−3^ for *a* = 5.95, 5.96, 5.97, respectively (corresponding to *D* values where peak max_*τ*_ *C*(*τ*) occurs in Fig. 14). For every value of *a*, correlation coefficient max_*τ*_ *C*(*τ*) exhibits high values for $2.0\times {10}^{-5}\lesssim {\rm{\Omega }}\lesssim 1.0\times {10}^{-3}$ and weak input signal $2.0\times {10}^{-2}\lesssim A\lesssim 8.0\times {10}^{-2}$. Compared to the chaotic resonance shown in Fig. 13, the sensitivity and degree of the signal response are lower.

Next, we investigate the relationship between input signal amplitude and signal response frequency. Figure 17 shows correlation max_*τ*_ *C*(*τ*) according to the amplitude and frequency of *S*(*t*) for various values of *D* and *a*, where *D* corresponds to peaks in correlation max_*τ*_ *C*(*τ*) (Fig. 14). High values of max_*τ*_ *C*(*τ*) $\gtrsim$ 0.3 are achieved in region $2.0\times {10}^{-5}\lesssim {\rm{\Omega }}\lesssim 1.0\times {10}^{-3}$ and $2.0\times {10}^{-2}\lesssim A\lesssim 8.0\times {10}^{-2}$ for every value of *a*.

## Discussion

To control chaotic resonance under a separating attractor into different regions, we propose the application of negative RRO feedback. This feedback was applied to a discrete neural system which is composed of excitatory and inhibitory neurons. As a result, it has been confirmed that negative RRO feedback can merge the separated attractor and induce chaotic resonance. For comparison on attractor merging, white Gaussian noise was also applied to the neural system, inducing stochastic resonance through attractor merging.

Regarding the characteristics of chaotic resonance induced by negative RRO feedback, around the attractor merging strength, the correlation between the effective neural potential and input signal as index of signal response exhibits a unimodal peak. This signal response is maximized at appropriate input signal amplitude and frequency. These characteristics are similar to those induced by positive RRO feedback^59^ and agree with those of chaotic resonance in other systems^36,37,39–41,43^.

Compared to stochastic resonance, additive noise and negative RRO feedback have the same effect for attractor merging and enhancing the signal response. However, the sensitivity against weak input signal in chaotic resonance is higher than that in stochastic resonance (see Figs 12, 13, 16 and 17). Besides high sensitivity, the degree of signal response in chaotic resonance is higher compared to stochastic resonance (see the peak values in the resonance regions of Figs 12, 13, 16 and 17, max_*τ*_ *C*(*τ*) ≈ 0.7 for chaotic resonance and max_*τ*_ *C*(*τ*) ≈ 0.4 for stochastic resonance). This higher ability of chaotic resonance than stochastic resonance agrees with our findings in the case where chaotic resonance is controlled by an internal system parameter^45,46^. Therefore, in chaotic resonance induced by negative RRO feedback, this higher sensitivity is maintained. Moreover, additive noise only merges the separated attractor, whereas RRO feedback can either merge or separate the attractor by applying negative or positive feedback, respectively (chaotic resonance under positive RRO feedback is detailed in our previous study^59^). Therefore, RRO feedback can be adopted for more varied attractor conditions compared to additive noise.

Some limitations of this study should be considered. The neural system proposed by Sinha is the simplest neuron model for eliciting chaos–chaos intermittency. Therefore, for applying the RRO feedback method to actual neural systems, chaotic resonance induced by RRO feedback must be evaluated in more realistic neural systems (i.e. complex, continuous, and high-dimensional neural networks). We are currently developing a method to apply RRO feedback in continuous chaotic systems utilizing the dynamics on Poincaré sections. This approach might be the suitable for applying RRO feedback to continuous and high-dimensional systems.

In this paper, we have reported that chaotic resonance can be induced by an external RRO feedback signal in neural systems without constraints on whether the attractor is merging or separating. Moreover, we confirm that chaotic resonance controlled by RRO feedback signal provides wider applicability than stochastic resonance. The outcomes of this study might promote the development of devices to strengthen signal responses by the effect of chaotic resonance in actual neural systems, where internal parameters cannot be controlled from outside the system.
